# Adapting the Technology Acceptance Model to Examine the Use of Information Communication Technologies and Loneliness Among Low-Income, Older Asian Americans: Cross-Sectional Survey Analysis

**DOI:** 10.2196/63856

**Published:** 2025-01-08

**Authors:** Pauline DeLange Martinez, Daniel Tancredi, Misha Pavel, Lorena Garcia, Heather M Young

**Affiliations:** 1 Betty Irene Moore School of Nursing University of California, Davis Sacramento, CA United States; 2 Department of Pediatrics University of California, Davis Sacramento, CA United States; 3 Khoury College of Computer Science Northeastern University Boston, MA United States; 4 Department of Public Health Sciences University of California, Davis Sacramento, CA United States

**Keywords:** social isolation, loneliness, aged, older adults, Asian American, immigrant, vulnerable populations, internet, information and communication technologies, ICTs, digital divide, technology acceptance model, mobile phone

## Abstract

**Background:**

Loneliness is a significant issue among older Asian Americans, exacerbated by the COVID-19 pandemic. Older age, lower income, limited education, and immigrant status heighten loneliness risk. Information communication technologies (ICTs) have been associated with decreased loneliness among older adults. However, older Asian Americans are less likely to use ICTs, particularly if they are immigrants, have limited English proficiency, or are low income. The Technology Acceptance Model posits that perceived usefulness (PU), and perceived ease of use (PEOU) are key factors in predicting technology use.

**Objective:**

This study aimed to examine associations between PU, PEOU, ICT use, and loneliness among low-income, older Asian Americans.

**Methods:**

Cross-sectional survey data were gathered from predominately older Asian Americans in affordable senior housing (N=401). Using exploratory factor analysis and Horn parallel analysis, we examined 12 survey items to identify factors accounting for variance in ICT use. We deployed structural equation modeling to explore relationships among the latent factors and loneliness, adjusting for demographic and cognitive factors.

**Results:**

Exploratory factor analysis and Horn parallel analysis revealed 3 factors that accounted for 56.48% (6.78/12) total variance. PEOU combined items from validated subscales of tech anxiety and comfort, accounting for a 28.44% (3.41/12) variance. ICT use combined years of technological experience, computer, tablet, and smartphone use frequency, accounting for 15.59% (1.87/12) variance. PU combined 2 items assessing the usefulness of technology for social connection and learning and accounted for a 12.44% (1.49/12) variance. The 3-factor structural equation modeling revealed reasonable fit indexes (χ^2^_133_=345.132; *P*<.001, chi-square minimum (CMIN)/df = 2595, comparative fit index (CFI)=0.93, Tucker-Lewis Index (TLI)=0.88). PEOU was positively associated with PU (β=.15; *P*=.01); PEOU and PU were positive predictors of ICT use (PEOU β=.26, *P*<.001; PU β=.18, *P*=.01); and ICT use was negatively associated with loneliness (β=–.28, *P*<.001). Demographic and health covariates also significantly influenced PU, PEOU, ICT use, and loneliness. English proficiency and education positively predicted PEOU (*r*=0.25, *P*<.001; *r*=0.26, *P*<.001) and ICT use (β=1.66, *P*=.03; β=.21, *P*<.001), while subjective cognitive decline and Asian ethnicity were positively associated with loneliness (β=.31, *P*<.001; β=.25, *P*<.001).

**Conclusions:**

This study suggests that targeted interventions enhancing PU or PEOU could increase ICT acceptance and reduce loneliness among low-income Asian Americans. Findings also underscore the importance of considering limited English proficiency and subjective cognitive decline when designing interventions and in future research.

## Introduction

### Background

The 2019 California Health Interview Survey (CHIS) found that 1 in 4 (25.7%) Asian Americans aged 65 years and older were lonely [1]. Loneliness is defined as a subjective experience stemming from perceived isolation or a disparity in one’s desired and actual social interactions [2]. In the CHIS, Asian American older adults reported significantly lower levels of perceived social and emotional support (56%) as compared with non–Asian American older adults (80%) [3]. During the COVID-19 pandemic, loneliness increased among older Americans, and even today, loneliness levels are higher as compared with prepandemic levels [4]. In 2023, the Surgeon General announced that the United States is experiencing a pandemic of loneliness [2].

As compared with younger generations, older adults are particularly at risk for loneliness and social isolation due to factors such as retirement, relocation, and shrinking social circles. Besides older age, other risk factors for loneliness include financial insecurity, low educational attainment, poor physical or mental health, being an immigrant, having a disability, and living alone [2,5-7]. A systematic review exploring factors associated with loneliness among older Asian American immigrants found that migration grief, diminished ethnic ties, mental and physical impairment, deteriorating health conditions, living alone, a lack of meaningful social connections and support networks, and fewer interactions with family members were all significant factors contributing to loneliness [8].

Among older adults, the use of information communication technologies (ICTs), including smartphones, tablets, personal computers, the internet, and social media, is associated with decreased loneliness [9,10]. ICT use can strengthen preexisting connections with family and friends, foster new social relationships, and build intergenerational bonds [9]. ICT use is also positively associated with self-efficacy, self-esteem, a sense of autonomy, independence, and greater well-being among older adults [9]. Older Asian Americans can further benefit from using ICTs to stay in contact with distant relatives and maintain a connection with their culture of origin; this is particularly relevant given that 85% of Asian Americans aged 65 years and older are foreign-born [11-13]. Furthermore, ICTs can facilitate access to information in one’s native language and translation services. However, both the CHIS and the National Health and Aging Trends Study (NHATS) showed that older Asian Americans are less likely than non-Hispanic White older adults to use the internet, send emails or text messages, conduct personal tasks on the internet, or seek web-based health information, particularly if they are immigrants, have limited English proficiency, or are low income [14-16]. Furthermore, other factors, such as age, gender, educational attainment, and subjective cognitive decline significantly impact ICT acceptance and use among low-income, older Asian Americans [17,18].

A recent systematic mapping review identified 59 articles describing 119 factors that predict older adults’ intention to use digital technologies [19]. However, despite a rich literature focused on this topic, the mapping revealed that most studies (68%) did not examine these factors using an established theoretical framework or model for technology acceptance. For example, although loneliness was identified as a factor associated with ICT use, it has not been analyzed through a theoretical framework or model [19].

### Theoretical Framework

The Technology Acceptance Model (TAM) is the most commonly used theoretical model to study technology adoption among older adults and also among the general population [19-21]. The backbone of the TAM is comprised of perceived usefulness (PU) and perceived ease of use (PEOU). PU relates to one’s perception of technology as being useful for accomplishing desired goals, while PEOU refers to how much effort one anticipates needing to make to learn to use new technology. The TAM proposes that PEOU predicts PU, PU, and PEOU predict attitudes toward technology, and these attitudes predict behavioral intention to use technology, which subsequently influences actual use [21].

In 2 previous studies, we validated a simplified TAM to predict ICT use among low income, older Asian Americans. The simplified model removed the mediators (attitudes toward technology and behavioral intentions) and adapted the constructs of PU and PEOU from the original TAM [17,18]. In the adapted model, PU was defined as older adults’ perceptions of ICTs as being useful for connecting with family and friends and learning new information and skills, and PEOU was operationalized using 6 evidence-based items that measure older adults’ comfort and anxiety with ICTs [18]. However, in our previous work, we had not empirically examined the assumptions of the operationalization of PU, PEOU, and ICT use using robust statistical methods. In addition, the association between ICT use and loneliness among older Asian Americans has never previously been examined using the TAM framework.

### Research Design

**Figure 1:**
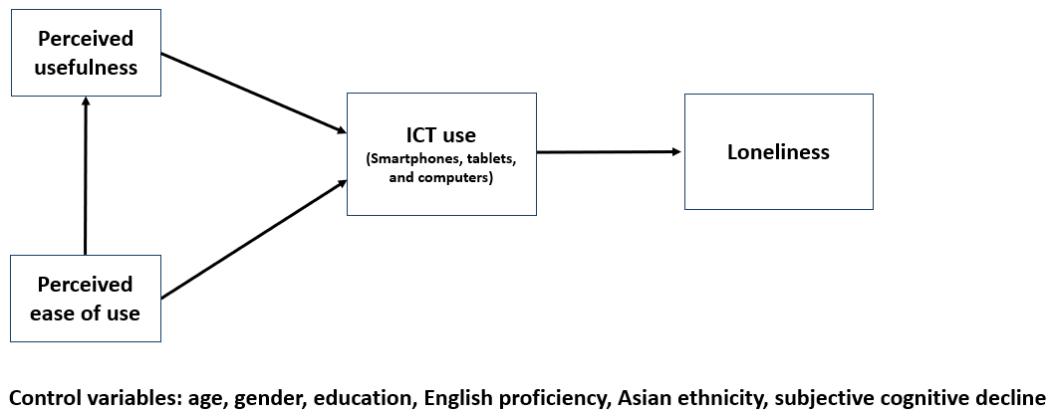
Extended Technology Acceptance Model examining the association between information communication technologies acceptance and loneliness among low income, older Asian Americans. ICT: information communication technology.

This cross-sectional study aimed to rigorously assess the assumptions underlying the conceptualization of PU, PEOU, and ICT use using a series of statistical techniques. In addition, we plan to extend the simplified TAM as described in DeLange Martinez et al [18] to explore the association between technology acceptance and loneliness among low income, older Asian Americans (Figure 1). Based on previous studies, we adjusted the model for age, gender, education, English proficiency, Asian ethnicity, and subjective cognitive decline [17,18].

In this study, we test three hypotheses, that are (1) H1: PEOU will be positively associated with PU. (2) H2: PEOU and PU will be significant, positive predictors of ICT use. (3) H3: ICT use will be significantly negatively associated with loneliness.

## Methods

### Data and Sample

In the Fall of 2020, The Lighthouse Project for Older Adults was launched with the aim of developing a scalable model to address barriers to technology use among residents of affordable senior housing communities. To inform the intervention, focus groups were held with 29 residents and 13 staff across 2 communities. The discussion revealed many challenges, including high rates of social isolation, low literacy levels in multiple languages, cognitive and sensory challenges, low comfort with technology, and lack of infrastructure [22].

To address these challenges, residents were offered access to high-speed broadband internet, ICT devices, a series of digital literacy training courses, and tech support led by peer ambassadors. As part of the program evaluation, participants completed pre- and postsurveys. The surveys were evidence-based, translated into 5 languages (English, Spanish, Chinese, Vietnamese, and Korean), and pilot-tested with 20 residents and 4 staff. The final surveys were self-administered by all Lighthouse participants in their preferred language with staff available to assist as needed.

### Measures

The operationalization of PU, PEOU, and ICT use were based on the theoretical constructs from the original TAM [21]. In 2 previous studies, we operationalized these 3 constructs with 12 survey items as described below, standardizing and summing the items for subsequent analysis with hierarchical linear regression [17,18]. In this study, we examined the theoretical assumptions of these constructs by using exploratory factor analysis (EFA), Horn parallel analysis, and structural equation modeling (SEM).

#### Perceived Usefulness

In the TAM, PU refers to whether one perceives technology to be useful for accomplishing desired goals [21]. In this study, we operationalized PU with 2 items developed by Sims et al [23] in their measure of Motivations for ICT Use. (“Technology helps me be connected with family and friends,” and “Technology helps me learn new information and skills.”) Response categories for both statements ranged on a scale of 1 (strongly disagree) to 4 (strongly agree).

#### Perceived Ease of Use

In the TAM, the construct of PEOU refers to how much effort one anticipates needing to make to learn to use a new technology. PEOU has been operationalized to measure feelings of confusion, frustration, or ease when using technology; predictability or intuitiveness of the system; and frequency of making mistakes [21]. In this study, we examined 6 items from 2 validated subscales that we predicted would collectively represent the construct of PEOU. Two items were included from the Senior TAM – Tech Anxiety Subscale [24] (“I feel apprehensive about using technology,” and, “I hesitate to use technology for fear of making mistakes I cannot correct.”) and 4 items were included from the Attitudes Towards Computers Questionnaire (ATCQ) – Comfort Subscale [25] (“I feel comfortable with technology” (reverse scored), “Technology makes me nervous,” “I don’t feel confident about my ability to use technology,” and, “Technology is confusing”). Response options for all statements ranged on a scale from 1 (strongly agree) to 4 (strongly disagree). Although the original ATCQ - Comfort Subscale included 5 items, one item was not included in the Lighthouse for Older Adults survey (“Computers make me feel dumb.”) based on input from staff about cultural relevance.

#### ICT Use

We operationalized ICT use with 4 survey items. Three items asked about the frequency of use of computers, tablets, and smartphones. (“How often do you use a desktop or laptop computer?”; “How often do you use a tablet or iPad?”; and “How often do you use a smartphone (iPhone or Android)?”) Response options ranged from 0 (never, or I do not own), to 3 (about once per day). A fourth item inquired about years of experience using ICTs (“How long have you been using technology, such as a computer, laptop, tablet, or smartphone?”). Response options ranged from 0 (I’ve never used these) to 3 (more than 2 years).

#### Dependent Construct: Loneliness

Loneliness was measured using the 3-item University of California, Los Angeles (UCLA) Loneliness Scale (“How often do you feel that you lack companionship?”; “How often do you feel left out?”; and “How often do you feel isolated from others?”) [26]. While the University of California, Los Angeles Loneliness Scale typically includes 3 response options (hardly ever, some of the time, often), a fourth answer option (never) was added because several residents handwrote “never” in the margins of the survey during pilot testing. During analysis, “never” responses were collapsed with “hardly ever.” Therefore, in our analysis, response categories ranged on a scale of 1 (never or hardly ever) to 3 (often).

#### Control Variables

In our final SEM, we controlled for age, gender, Asian ethnicity, education, subjective cognitive decline (measured with 1 item, “During the past 12 months, have you experienced confusion or changes in memory that is happening more often or is getting worse?” [dichotomous response options]), and English proficiency (measured with one item, “How well do you speak English?” (response options ranged from 1 [not at all] to 4 [very well]). The full survey used in the Lighthouse Project for Older Adults is attached as a Multimedia Appendix 1.

### Analytic Strategy

We began our analysis by examining descriptive statistics and conducting Pearson correlation analysis to explore relationships among all variables.

Subsequently, using IBM SPSS Statistics (version 29), we performed EFA to investigate the underlying theoretical constructs of the survey items concerning attitudes and use of ICTs. Despite having hypotheses about the latent variables, we chose to conduct EFA before confirmatory factor analysis (CFA), aiming for a more data-driven approach to thoroughly explore the underlying structure. To determine the number of factors to retain, we applied several criteria, including factors with loadings greater than .45 and using Kaiser eigenvalues-greater-than-one rule as illustrated on a scree plot [27]. We used principal axis factoring and varimax rotation with Kaiser normalization to account for the correlational nature of the factors. In addition, Horn parallel analysis was used to confirm the EFA results.

Moving forward, we used IBM SPSS AMOS (version 29) to conduct SEM, beginning with CFA. SEM integrates measurement models and structural models, allowing for validation of instruments and analysis of relationships while considering variances and covariances. It facilitates the examination of complex relationships among multiple variables and enables mediation analysis [28]. During CFA, we assessed convergent validity using criteria suggested by Hair et al [29] and Fornell and Larcker [30], calculating average variance extracted (AVE) and composite reliability. Discriminant validity was determined based on the Fornell-Larcker criterion [30].

Next, we used SEM to represent and test our 3 hypotheses, exploring relationships among PEOU, PU, ICT use, and our outcome of interest (loneliness). As shown in Figure 1, we examined a model with PU partially mediating the relationship between PEOU and ICT use, and ICT use fully mediating the relationships between PU, PEOU, and loneliness. We also examined the impact of adjusting for demographic and cognitive factors based on previous findings [15-18]. Maximum likelihood estimation was used for factor structure verification, and missing data were imputed by estimating means and intercepts. Measures of fit, including chi-square statistic (*χ*^2^), chi-square divided by degrees of freedom (*χ*^2^/df), comparative fit index (CFI), Tucker-Lewis coefficient (TLI), and root-mean-square error of approximation (RMSEA), were reported. Regression weights and correlation estimates among latent factors, the outcome of interest, and control factors were also provided. For all analyses, the α-level for testing significance was set to .05.

### Ethical Considerations

For this study, we analyzed presurveys from 5 Lighthouse communities, collected between July 2021 and July 2022, before receiving the intervention. All participants were aged 62 years and older, based on housing eligibility criteria. In total, 31 participants were excluded from the analysis due to missing at least 1 of the dependent variables. The final dataset included 401 participants. On the basis of the HRP-210 Determination Request, the University of California, Davis, institutional review board determined that this research is exempt as it did not directly involve human participants and used deidentified secondary data (ID: 1938286-1).

## Results

### Overview

Participant (n=401) demographics are described in Table 1. Participants ranged in age from 62 to 97 (mean 79.07, SD 7) years, most were female, had a high school degree or less, reported limited English proficiency, and were Asian. Over a quarter of participants reported subjective cognitive decline and over a third reported loneliness.

**Table 1:** Participant demographics (N=401).

Participant demographics		Frequency, n	Percentage, %
**Gender**			
	Female	276	69.2
	Male	123	30.8
**Ethnicity**			
	Non-Hispanic White	48	12.0
	Asian	320	79.8
	Korean	262	65.3
	Chinese	50	12.5
	Japanese	2	0.5
	Filipino	2	0.5
	Vietnamese	1	0.2
	Latinx	11	2.7
	Black or African American	6	1.5
	American Indian or Alaskan Native	2	0.5
	More than one race or ethnicity	7	1.7
**English proficiency**			
	Very well	58	14.8
	Well	62	15.8
	Not well	172	43.8
	Not at all	101	25.7
**Educational attainment**			
	Never attended school	21	5.5
	Some high school	127	33.0
	Completed high school or general educational development	72	18.7
	Some college	75	19.5
	College degree	65	16.9
	Graduate degree	25	6.5
**Subjective cognitive decline**			
	No	283	72.2
	Yes	109	27.8
**Years of experience using** **information communication technologies**			
	More than 2 years	239	59.6
	1 to 2 years	39	9.7
	Less than 1 year	32	8.0
	I have never used these	91	22.7
**Computer use**			
	About once per day	87	21.7
	2 to 4 times per week	28	7.0
	Once or less than once per week	25	6.2
	Never	261	65.1
**Tablet use**			
	About once per day	92	22.9
	2 to 4 times per week	29	7.2
	Once or less than once per week	25	6.2
	Never	255	63.6
**Smartphone use**			
	About once per day	247	61.6
	2 to 4 times per week	39	9.7
	Once or less than once per week	16	4.0
	Never	99	24.7

About 1 in 5 participants reported they have never used a smartphone, tablet, or computer, while 6 in 10 reported over 2 years of experience using these devices. When asked about the frequency of use of each type of device, two-thirds reported that they never use a computer, slightly less than two-thirds never use a tablet, and a quarter never use a smartphone. Despite this, 89.2% (340/381) somewhat or strongly agreed that technology is useful for connecting with family and friends, and 90.4% (341/377) agreed that technology is useful for learning new information and skills.

Next, we used correlation analysis to examine relationships among items measuring ICT use, PEOU, PU, loneliness, and our control variables (age, gender, Asian ethnicity, education, subjective cognitive decline, and English proficiency). The assumptions for factor analysis were met. We observed multiple correlations among the items, most ranging from 0.30 and above. Importantly, none of these correlations exceeded 0.9, indicating no issues with multicollinearity. Correlation results are included in Multimedia Appendix 2.

### EFA and Horn Parallel Analysis

The Kaiser-Meyer-Olkin measure of sampling adequacy was .83 indicating sufficient correlation among the variables. The Bartlett test of Sphericity indicated *P*<.001, allowing us to reject the null hypothesis that the correlation matrix is an identity matrix and that it is reasonable to proceed with EFA.

**Figure 2:**
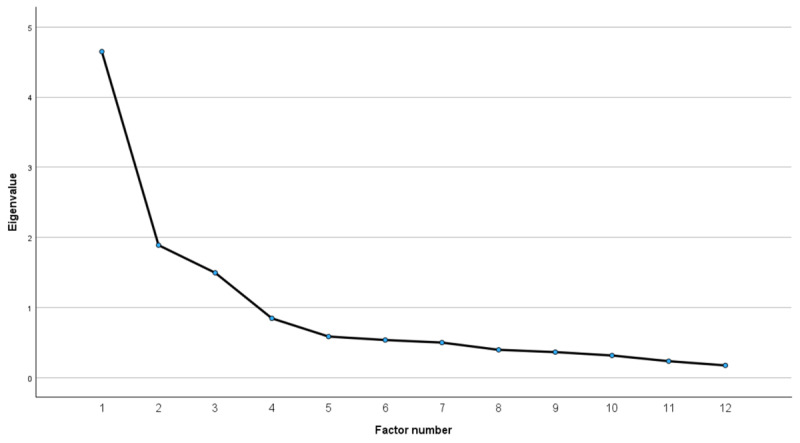
Scree plot.

Three factors had an initial eigenvalue greater than one (Factor 1=4.65, Factor 2=1.89, and Factor 3=1.50). This is illustrated with the scree plot (Figure 2). The rotated factor matrix is shown in Table 2.

**Table 2:** Rotated factor matrix of independent variables.

Variables	Factor		
	PEOU^a^	ICT^b^ use	PU^c^
Tech comfort	.40	.42	.35
Tech nervous	.72	.08	.01
Tech confidence	.82	.16	.10
Tech confusion	.88	.14	.08
Tech apprehension	.79	.20	.10
Tech fear	.75	.22	.10
Tech connection	.06	.07	.75
Tech learning	.09	.15	.85
Years of tech experience	.21	.60	.16
Computer use frequency	.19	.67	.03
Tablet use frequency	.03	.68	–.03
Smartphone use frequency	.11	.52	.17

^a^PEOU: perceived ease of use.

^b^ICT: Information communication technology.

^c^PU: perceived usefulness.

The first factor, PEOU, combined the 2 items from the Senior TAM – Tech Anxiety Subscale and 3 of the 4 items from the Attitudes Towards Computers Questionnaire – Comfort Subscale. One item, “I feel comfortable with technology,” had a factor loading of .40. According to Tabachnick and Fidell [27], loading above 0.71 is excellent, 0.63 is very good, 0.55 is good, 0.45 fair, and 0.32 poor. Therefore, we dropped the tech comfort item from PEOU in further analyses.

The second factor, ICT use, combined 4 items: years of tech experience, computer use frequency, tablet use frequency, and smartphone use frequency.

The third factor, PU, combined 2 items: “Technology helps me be connected with family and friends,” and “Technology helps me learn new information and skills.”

After rotation, the 3 factors combined accounted for a total variance of 56.48% (6.78/12), with PEOU accounting for 28.44% (3.41/12) of the variance, ICT use for 15.59% (1.87/12), and PU for 12.44% (1.49/12). The 3 factors were confirmed when running a Horn parallel analysis. These factors also showed high internal reliability with Cronbach α scores of .90, .74, and .76 for PEOU, ICT use, and PU, respectively.

EFA was conducted separately for the dependent variables and generated 1 interpretable factor, loneliness, with an eigenvalue of 2.29 (Table 3). Loneliness had strong internal reliability with a Cronbach α score of .84.

**Table 3:** Component matrix for dependent variables using principal extraction method.

Variables	Factor (loneliness)
Lack companionship	.69
Feel left out	.93
Feel isolated	.79

### CFA Findings

**Figure 3:**
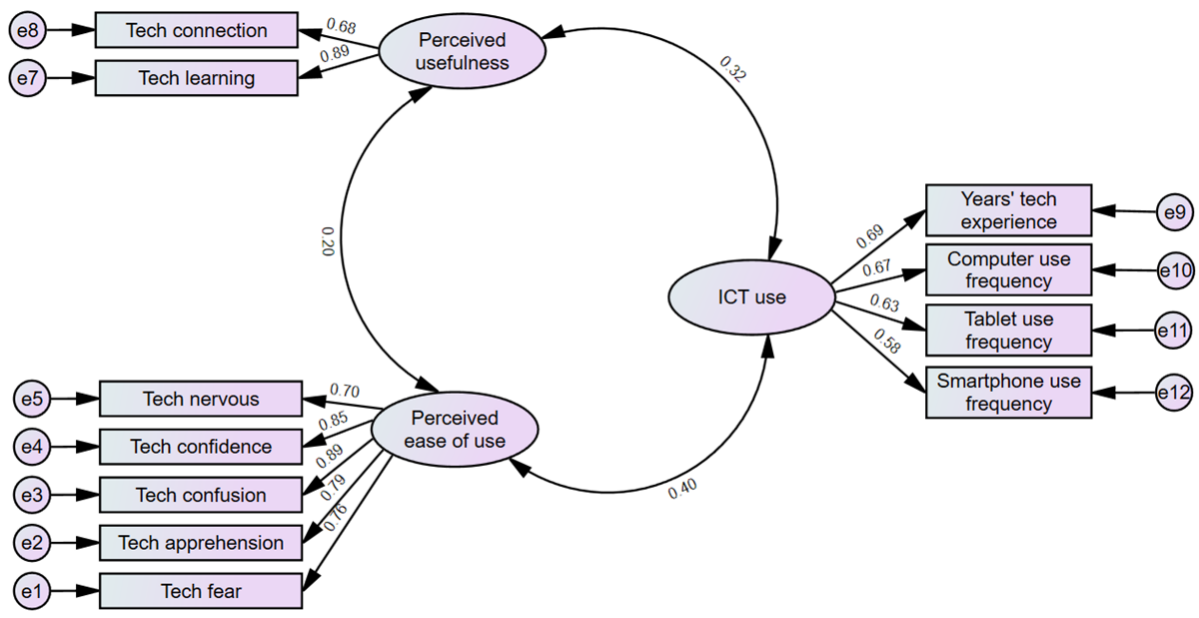
Standardized results from confirmatory factor analysis to assess our conceptual model. ICT: information communication technology.

We ran CFA to further examine the relationships among the latent variables and to assess our conceptual model (Figure 3). As shown in Table 4, each of the items significantly loaded to form the 3 latent factors, confirming our EFA results.

Except for chi-square, all fit indices reached recommended level of fit: (*χ*²_41_=182.114; *P*<.001, chi-square minimum (CMIN)/df=4.44, CFI=0.92, TLI=0.87). The RMSEA of 0.09 was borderline. Since *χ*² is sensitive to large sample sizes, with a large sample of 401 participants, it was not unusual to get a significant value; for sample sizes greater than 250, a significant *χ*² value is acceptable [31].

**Table 4:** Regression estimates of items loading into 3 factors.

Observed variables		Latent variables	Standardized regression estimate (factor loading)	Unstandardized regression estimate	SE	Composite reliability	*P* value
Tech fear	←	PEOU^a^	.76	1.00	—^b^	—	—
Tech apprehension	←	PEOU	.80	.99	.06	15.963	<.001
Tech confusion	←	PEOU	.89	1.09	.06	18.156	<.001
Tech confidence	←	PEOU	.85	1.05	.06	17.130	<.001
Tech nervous	←	PEOU	.70	.86	.06	13.797	<.001
Tech learning	←	PU^c^	.90	1.00	—	—	—
Tech connection	←	PU	.68	.77	.16	4.796	<.001
Years of tech experience	←	ICT^d^ use	.69	1.00	—	—	—
Computer use frequency	←	ICT use	.67	.97	.10	10.053	<.001
Tablet use frequency	←	ICT use	.63	.93	.10	9.719	<.001
Smartphone use frequency	←	ICT use	.58	.86	.09	9.166	<.001

^a^PEOU: perceived ease of use.

^b^Not applicable.

^c^PU: perceived usefulness.

^d^ICT: Information communication technology.

There was evidence for convergent validity because all three of the conditions were fulfilled, that is, (1) composite reliability values are 0.7 or greater, (2) all standardized factor loadings are 0.5 or greater, and (3) AVE values are 0.5 or greater [29]. All 3 of these criteria were met for PEOU and PU, which had composite reliability values of .90 and .77, standardized factor loadings all greater or equal to .68, and AVE values of .64 and .63, respectively.

ICT use had an AVE of .42, slightly lower than ideal [29]. However, according to Fornell and Larcker [30], if the AVE is less than 0.5, but composite reliability is higher than 0.6, the convergent validity of the construct is acceptable [30]. ICT use had a composite reliability of .74, therefore we concluded that the latent variable had convergent validity.

Discriminant validity was met, with discriminant values of .80, .80, and .65 for PEOU, PU, and ICT use, respectively, while the correlation estimates all fell below 0.4 as shown in Table 5 [30].

Finally, as shown in Table 6, the relationships between the latent variables were all significant.

**Table 5:** Correlations and covariances of latent variables in confirmatory factor analysis.

Latent variables			Correlation estimate	Covariance estimate	SE	Composite reliability	*P* value
PEOU^a^	↔	PU	.200	.107	.033	3.273	.001
ICT^b^ Use	↔	PU	.317	.190	.042	4.501	<.001
ICT Use	↔	PEOU	.399	.269	.048	5.643	<.001

^a^PEOU: perceived ease of use.

^b^ICT: Information communication technologies.

**Table 6:** Regression weights (unadjusted model).

Latent variables			Standardized regression estimate	Unstandardized regression estimate	SE	Composite reliability	*P* value
PU^a^	←	PEOU	.20	.18	.05	3.38	<.001
ICT^b^ Use	←	PU	.24	.31	.10	3.19	.001
ICT Use	←	PEOU	.37	.41	.07	5.75	<.001
Loneliness	←	ICT Use	-.29	-.15	.03	-4.48	<.001

^a^PU: perceived usefulness.

^b^ICT: Information communication technologies.

### Structural Equation Modeling

The 3-factor SEM revealed reasonable fit indexes (χ^2^_73_=231.835, CMIN/DF=3.18, CFI=0.93, TLI=0.90). An RMSEA of 0.07 was acceptable. Once again, the chi-square value was significant, which, as previously described, is acceptable for this sample size [31].

**Figure 4:**
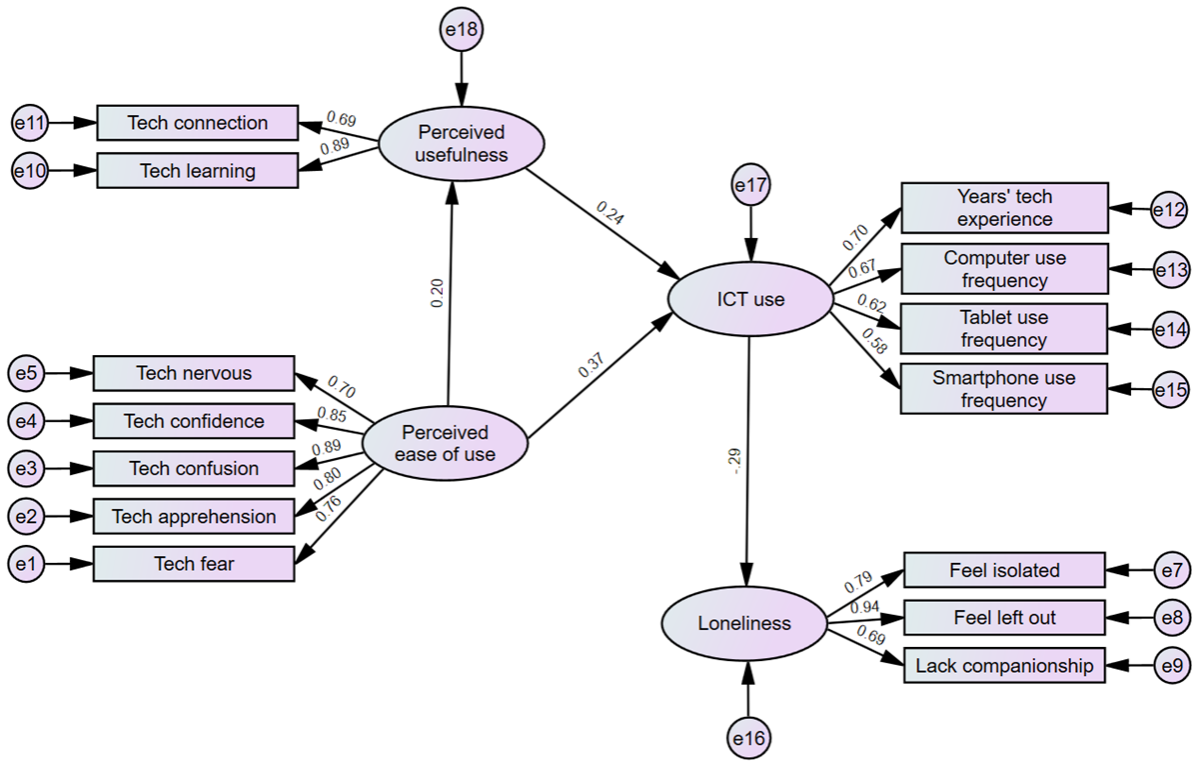
Standardized results from structural equation modeling (unadjusted model).

As shown in Figure 4 and Table 6, hypothesis 1 was supported; PEOU was significantly, and positively associated with PU (β=.20, *P*<.001).

Hypothesis 2 was also supported; both PEOU and PU were significant, positive predictors of ICT use (PEOU: β=.37; *P*<.001; PU: β=.24; *P*=.001).

Finally, hypothesis 3 was supported; ICT use was significantly, negatively associated with loneliness (β=–.29; *P*<.001).

### Structural Equation Modeling Adjusting for Control Factors

**Figure 5:**
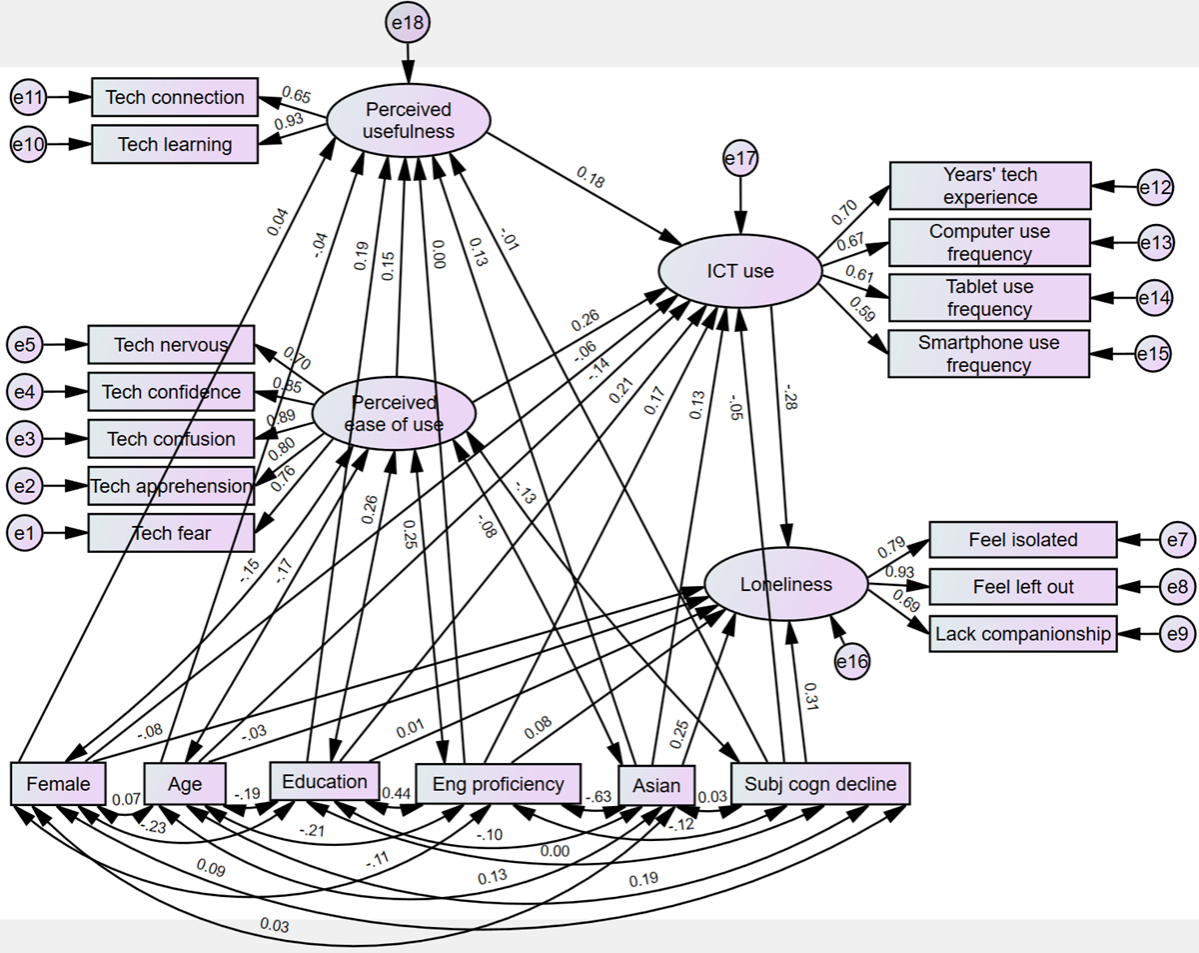
Adjusted, standardized results from structural equation modeling. ICT: information communication technology.

When we reran the model adjusting for age, gender, education, Asian ethnicity, English proficiency, and subjective cognitive decline, the model fit improved (Figure 5; χ^2^_133_=345.13, *P*<.001, CMIN/DF=2595, CFI=0.93, TLI=0.88). The RMSEA of 0.06 was acceptable.

As shown in Table 7, the adjusted results continued to support Hypotheses 1, 2, and 3. PEOU continues to be significantly positively associated with PU (β=.152, *P*=.01). PEOU and PU were significant, positive predictors of ICT use (PEOU: β=.26, *P*<.001; PU: β=.179, *P*=.01). And, ICT use was significantly negatively associated with loneliness (β=–.28, *P*<.001). In addition, some of the control variables were significant predictors of the endogenous variables, PU and ICT use, and the dependent variable, loneliness (Table 7). Education was significantly, positively associated with PU (β=.19; *P*=.003). English proficiency and education significantly, positively predicted ICT use (English proficiency: β=1.66; *P*=.03; Education: β=.21; *P*<.001), while age was negatively associated with ICT use (β=–1.36; *P*=.01). Finally, subjective cognitive decline and Asian ethnicity were each positively associated with loneliness (subjective cognitive decline: β=.31; *P*<.001; Asian ethnicity: β=.25; *P*<.001).

**Table 7:** Regression weights (adjusted model).

Variables	Standardized regression estimate	Unstandardized regression estimate	SE	Composite reliability	*P* value
PU^a^←PEOU^b^	.15^c^	.14	.06	2.55	.01
PU←English proficiency	–.003	–.002	.06	–.04	.97
PU←Asian	.13	.24	.13	1.83	.07
PU←subjective cognitive decline	–.01	–.02	.09	–.22	.83
PU←age	–.04	–.004	.01	–.78	.44
PU←education	.19^c^	.10	.03	2.98	.003
PU←female	.04	.07	.09	.80	.42
ICT^d^ use←PU	.18^c^	.22	.08	2.70	.01
ICT use←female	–.06	–.12	.10	–1.17	.24
ICT use←age	–.14^c^	–.02	.01	–2.52	.01
ICT use←English proficiency	.17^c^	.15	.07	2.13	.03
ICT use←subjective cognitive decline	–.05	–.10	.10	–.92	.36
ICT use←PEOU	.26^c^	.29	.07	4.30	<.001
ICT use←education	.21^c^	.13	.04	3.30	<.001
ICT use←Asian	.13	.29	.15	1.93	.05
Loneliness←female	–.08	–.08	.05	–1.59	.11
Loneliness←age	–.03	–.002	.003	–.63	.53
Loneliness←English proficiency	.08	.04	.04	1.01	.31
Loneliness←ICT use	–.28^c^	–.14	.04	–3.93	<.001
Loneliness←subjective cognitive decline	.31^c^	.31	.05	5.76	<.001
Loneliness←education	.01	.004	.02	.21	.84
Loneliness←Asian	.25^c^	.28	.08	3.64	<.001

^a^PU: perceived usefulness.

^b^PEOU: perceived ease of use.

^c^*P*≤.05.

^d^ICT: Information communication technology.

While PEOU was an exogenous factor, correlation estimates reveal significant associations with all the control variables except for Asian ethnicity (Table 8). English proficiency and education were each significantly positively associated with PEOU (English proficiency: *r*=0.25, *P*<.001; Education: *r*=0.26; *P*<.001). Subjective cognitive decline, age, and female gender were each significantly, and negatively associated with PEOU (Subjective cognitive decline: *r*=–0.130; *P*=.02; Age: *r*=–0.18; *P*=.001; Female: *r*=–0.15; *P*=.01).

**Table 8:** Correlations and covariances of control variables with perceived ease of use (adjusted model).

Control variables	Correlation estimate	Covariance estimate	SE	Composite reliability	*P* value
Asian	–.09	–.03	.02	–1.58	.11
Subjective cognitive decline	–.13	–.05	.02	–2.40	.02
English proficiency	.25	.19	.04	4.47	<.001
Education	.26	.28	.06	4.54	<.001
Age	–.18	–.95	.30	–3.18	.001
Female	–.15	–.05	.02	–2.73	.01

## Discussion

### Principal Findings

In this study, we examined the association of PU, PEOU, ICT use, and loneliness among low income, predominately Asian American, older adults. This research built upon 2 previous studies that simplified the TAM to examine demographic and health factors that impact technology acceptance among Asian Americans aged 62 years and older living in affordable senior housing communities [17,18]. In these previous analyses, the conceptualization of PU, PEOU, and ICT use were based on the existing literature and theory around how these constructs apply to older adults. The constructs were developed by summing and normalizing 12 survey items. In this study, the assumptions underlying the conceptualization of PU, PEOU, and ICT use and the TAM framework were rigorously assessed and confirmed using a combination of statistical techniques. Initially, EFA was conducted to determine the appropriate number of factors and explore the underlying structure of the constructs. Horn parallel analysis was subsequently used to validate the EFA results. Following this, SEM (beginning with CFA) was performed to validate and confirm the factor structure identified in EFA and examine the relationships among PU, PEOU, ICT use, and loneliness.

The original TAM focused on technology acceptance in the workplace. PU was measured with items such as, “Using [chart master] in my job would enable me to accomplish tasks more quickly,” and, “Using [chart master] would improve my job performance” [21]. Our research findings support a modified construct of PU that accounts for older adults’ perceptions of ICTs as being useful for social connection and for learning new information and skills. We found that the item, “Technology helps me learn new information and skills,” had a slightly higher factor loading than the item, “Technology helps me be connected with family and friends.” Mixed methods longitudinal data from the Lighthouse Project for Older Adults supported this finding; participants reported that they most frequently used ICTs to access YouTube (eg, to view videos related to nutrition, exercise, and cultural content), followed by accessing entertainment and checking the weather [32]. The Pew Research Center reported that, in 2021 among adults aged 65 years and older, the use of YouTube experienced the most growth as compared with any other app [33]. Notably, studies suggest that app- and web-based activities among older adults vary significantly by age group and gender [23,34-36].

When it comes to PU, it is essential to note that the COVID-19 pandemic spurred an exponential increase in the use of ICTs for social connection. American Association of Retired Persons (AARP) 2021 Tech Trends reported a notable surge in the use of various communication technologies among individuals aged 50 years and above to stay connected with others. A significant portion of this demographic reported an uptick in their usage of video chats (45%, 1022/2271), texting (37%, 840/2271), emailing (26%, 590/2271), and phone calls (29%; 659/2271) compared with prepandemic levels. In 2019, approximately half had never used video chat, whereas by 2020, this figure rose to 70%, with one out of 3 engaging in video chats on a weekly basis [37].

Our findings supported the operationalization of PEOU combining 2 items from the Senior TAM – Tech Anxiety Subscale [24] and 3 items from the ATCQ – Comfort Subscale [25]. Previous studies note an array of emotions that influence technology acceptance and use, including enjoyment, effort expectancy [38], control, efficacy [24,39], confidence [40,41], comfort [25,42,43], and anxiety [24,40]. Due to the variety of existing measures and constructs highlighted in the literature (some developed in the 1980s and with highly educated, White, middle-aged adults), it can be challenging for researchers to select a concise set of items that are specific to older adults and modern technology. We believe our findings can inform future studies with Asian American older adults, who require survey modification [44]. During exploratory analysis, one item, “I feel comfortable with technology,” was dropped due to low factor loading. We believe this may have been due to the item being reverse scored, which may have been confusing due to participants’ limited literacy levels. Our findings align with previous research which suggests that assessment scales containing reverse-scored items impose higher cognitive processing demands, potentially resulting in measurement challenges for older adult participants [45], particularly since our participants had self-reported limited English proficiency and low educational attainment (Table 1).

Our mediating factor, ICT use, was unique in that it combined measures assessing the frequency of use of smartphones, tablets, and computers, as well as years of experience. We believe this measure is valuable to better understand ICT use since these devices are often used interchangeably among older adults for multitasking [46]. Furthermore, we are not the first to identify years of experience as an important factor in understanding technology acceptance [38].

Using CFA, we found significant, positive relationships between PU, PEOU, and ICT use. This finding is aligned with the Senior Technology Exploration, Learning, and Acceptance model [47] and the Senior Technology Acceptance and Adoption Model (STAM) [48]. The concept of reinforcement of use is consistent with a model of technology acceptance or rejection from an ease-of-learning perspective [49]. When ICTs are perceived as more useful, their usage tends to increase, forming a reinforcing cycle. Increased usage and familiarity make it easier, encouraging individuals to master new skills and diversify their usage, such as progressing from social chats to watching YouTube videos, further reinforcing their mastery and use.

SEM confirmed our modified TAM, supporting our three hypotheses. PEOU significantly, and positively predicted PU and ICT use. Further, ICT use was significantly negatively associated with loneliness. These relationships remained significant, even when adjusting for gender, age, education, English proficiency, Asian ethnicity, and subjective cognitive decline. Interestingly, English proficiency was related to PEOU and ICT use, suggesting a cultural or linguistic bias in technology and application development favoring English-speaking users. Asian ethnicity was associated with loneliness, affirming observations of the vital role technology can play for immigrants in maintaining social and cultural connections [11]. Subjective cognitive decline was also associated with loneliness, perhaps a reflection of lower social and technology engagement [50].

This study has implications for interventions, particularly among populations with lower literacy. Both PEOU and PU are modifiable factors and could be enhanced by offering digital literacy training and support to new learners [43,51-53] or tailoring user interfaces [54,55]. The relevance of technology for the individual can be increased by demonstrating various culturally relevant use cases for devices, such as connecting with distant relatives, accessing health information, or enjoying entertainment in one’s native language. In the Lighthouse Project for Older Adults, the low-income housing providers made a commitment to enhance technology use and offered the technology, training, and support to encourage adoption. This not only provided tangible support in the form of equipment and training but also created a community of learners where individuals could benefit from the experience of peers [32]. This is an example of how service providers can play a role in potentially improving quality of life and reducing loneliness by encouraging the adoption of technology.

### Study Limitations

This study was partially limited by the use of a convenience sample of participants who were interested in learning more about technology, not a random sample. This potentially biases the sample toward those with more positive attitudes toward technology. Even with this bias, we observed variability in perceptions about technology but did not capture the full array of attitudes likely present in the population. In addition, participants all lived in age-restricted affordable housing communities. Therefore, our findings are not representative of older adults living in multigenerational households. We removed participants whose data were missing the dependent variable, loneliness. We retained all other participants, yet 4%-7% of responses were missing for items related to PEOU and PU.

Methodological considerations included minimizing participant burden to maximize potential engagement with technology and the most complete dataset. This required modifications to measures, to accomplish parsimony and ease of administration, potentially compromising psychometric properties. However, our CFA indicated the adequacy of the measures for crucial constructs, despite having fewer than 3 observations for each latent variable in the case of PU.

EFA and SEM were limited by the inclusion of binary and ordinal observed variables (eg, gender, Asian ethnicity, and subjective cognitive decline). While this represents a methodological weakness, these variables were included because of their known importance to the constructs of interest. Furthermore, we used the same dataset for all multivariate analyses because of the early phase of understanding relationships among our variables of interest, recognizing that typically the confirmatory analysis should be conducted on an independent sample.

Finally, many other factors potentially impact technology acceptance among older adults, including social and health factors, access to Wi-Fi and devices, and digital literacy training.

### Conclusion

Despite the limitations, this study affirms the usefulness of the TAM in understanding the dynamics of technology adoption among a low-income Asian American population. At baseline, there is considerable interest in technology, affirming its relevance in the lives of older adults. The role of English proficiency in ICT use warrants further exploration to identify ways to increase equity and access for those who have another primary language. The study further highlights the potential role that technology could play in alleviating loneliness through greater engagement with family and friends and the ability to maintain cultural ties. Future studies could explore the most effective ways to overcome resistance to technology, the most meaningful ways to support novice users to adopt a new device, and ways to increase the diversity of use once a user has become comfortable with basic functions. While technology cannot replace the human touch, it has the promise to improve engagement and social connection among isolated, low-income older adults.

## Appendix

Multimedia Appendix 1 Lighthouse survey.

Multimedia Appendix 2 Correlation matrix.

## Abbreviations

ATCQ: Attitudes Towards Computers Questionnaire
AVE: average variance extracted
CFA: confirmatory factor analysis
CFI: Comparative Fit Index
CHIS: California Health Interview Survey
CMIN: chi-square minimum
EFA: exploratory factor analysis
ICT: Information communication technology
NHATS: National Health and Aging Trends Study
PEOU: perceived ease of use
PU: perceived usefulness
RMSEA: root-mean-square error of approximation
SEM: structural equation modeling
STAM: Senior Technology Acceptance and Adoption Model
TAM: Technology Acceptance Model
TLI: Tucker-Lewis Index

## Data Availability

The datasets generated during and analyzed during this study are available from the corresponding author on reasonable request.
