# Supplementary material for: Adapting the Technology Acceptance Model to Examine the Use of Information Communication Technologies and Loneliness Among Low-Income, Older Asian Americans: Cross-Sectional Survey Analysis
Source: JMIR Aging. 2025 Jan 8;8:e63856. doi: 10.2196/63856 (PMC11754985; doi:10.2196/63856)
Supplement: Multimedia Appendix 1 [file aging_v8i1e63856_app1.docx]

**Thank you for participating in the Lighthouse Project**

**Please take a few minutes to fill out this survey by completing ALL of the questions and return it to [COLLECTOR] by [DATE].** If you need help completing this survey, please contact [**COLLECTOR**]. Your answers will be kept confidential. No one will see this information except the staff involved in this project. Please provide as much information as possible. Please darken the appropriate box for each question.

**First Name ___________________ Last Name ____________________**

1. **What is your year of birth?**

19

1. **What is your gender?**

| - Male | - Female | - Other (Please specify): _____________ |
| --- | --- | --- |

1. **Which of the following best describes you?**

| - American Indian or Alaskan Native - Asian - Native Hawaiian or Pacific Islander - Black or African American - Filipino | - Hispanic or Latino - White or Caucasian - Multiracial or biracial - Other: ______________ |
| --- | --- |

1. **What language(s) do you prefer to use to communicate? (check all that apply)**

| - English - Korean - Mandarin - Cantonese - Vietnamese | - Russian - Spanish - American Sign Language - Other: ______________ |
| --- | --- |

1. **How well do you speak English?**

| - Very well | - Well | - Not Well | - Not at All |
| --- | --- | --- | --- |

1. **What is your marital status?**

| - Married/Living with Partner - Widowed - Divorced | - Separated - Single |
| --- | --- |

1. **What is your highest level of education that you have completed?**

| - Never Attended School - Some High School - Completed High School or GED | - Some College - College Degree - Graduate Degree |
| --- | --- |

1. **In what country did you complete your highest level of school?**

| - US | - Outside of the US |
| --- | --- |

1. **In general, how would you rate your physical health?**

| - Excellent | - Very Good | - Good | - Fair | - Poor |
| --- | --- | --- | --- | --- |

1. **I have challenges in the following areas (check all that apply):**

| - Vision | - Hearing | - Mobility | - Other: __________________ | - None |
| --- | --- | --- | --- | --- |

1. **During the past 12 months, have you experienced confusion or changes in memory that is happening more often or is getting worse?**

| - Yes | - No |
| --- | --- |

1. **In general, how would you rate your emotional health?**

| - Excellent | - Very Good | - Good | - Fair | - Poor |
| --- | --- | --- | --- | --- |

1. **In the past 2 weeks, how often have you been bothered by:**

**Little interest or pleasure in doing things?**

| - Not at all (Never) | - Several days | | - More than half the days | | - Nearly every day | |
| --- | --- | --- | --- | --- | --- | --- |
|  | |  | |  | |  |

**Feeling down, depressed, or hopeless?**

| - Not at all (Never) | - Several days | - More than half the days | - Nearly every day |
| --- | --- | --- | --- |

1. **How often do you feel that you lack companionship?**

| - Never | - Hardly Ever | - Some of the Time | - Often |
| --- | --- | --- | --- |

1. **How often do you feel left out?**

| - Never | - Hardly Ever | - Some of the Time | - Often |
| --- | --- | --- | --- |

1. **How often do you feel isolated from others?**

| - Never | - Hardly Ever | - Some of the Time | - Often |
| --- | --- | --- | --- |

**FAMILY: Considering the people to whom you are related by birth, marriage, or adoption…**

1. **How many relatives do you see or hear from at least once a month?**

| - None | - 1 | - 2 | - 3 or 4 | - 5 to 8 | - 9 or more |
| --- | --- | --- | --- | --- | --- |

1. **How many relatives do you feel at ease with that you can talk about private matters?**

| - None | - 1 | - 2 | - 3 or 4 | - 5 to 8 | - 9 or more |
| --- | --- | --- | --- | --- | --- |

1. **How many relatives do you feel close to such that you could call on them for help?**

| - None | - 1 | - 2 | - 3 or 4 | - 5 to 8 | - 9 or more |
| --- | --- | --- | --- | --- | --- |

**FRIENDSHIPS: Considering all of your friends including those who live in your neighborhood and community…**

1. **How many of your friends do you see or hear from at least once a month?**

| - None | - 1 | - 2 | - 3 or 4 | - 5 to 8 | - 9 or more |
| --- | --- | --- | --- | --- | --- |

1. **How many friends do you feel at ease with that you can talk about private matters?**

| - None | - 1 | - 2 | - 3 or 4 | - 5 to 8 | - 9 or more |
| --- | --- | --- | --- | --- | --- |

1. **How many friends do you feel close to such that you could call on them for help?**

| - None | - 1 | - 2 | - 3 or 4 | - 5 to 8 | - 9 or more |
| --- | --- | --- | --- | --- | --- |

1. **How long have you been using technology, such as a computer, laptop, tablet or smartphone?**

| - More than 2 years | - 1 to 2 years | - Less than 1 year | - I have never used these |
| --- | --- | --- | --- |

1. **How often do you use a desktop or laptop computer?**

| □ About once per day | □ 2 to 4 times per week | □ Once or less than once per week | - Never | - I do not own a desktop or laptop |
| --- | --- | --- | --- | --- |

1. **How often do you use a tablet or iPad?**

| □ About once per day | □ 2 to 4 times per week | □ Once or less than once per week | - Never | - I do not own a tablet or iPad |
| --- | --- | --- | --- | --- |

1. **How often do you use a smartphone (iPhone or Android)?**

| □ About once per day | □ 2 to 4 times per week | □ Once or less than once per week | - Never | - I do not own a smartphone |
| --- | --- | --- | --- | --- |

1. **How often do you use an Amazon Alexa or Google Home?**

| □ About once per day | □ 2 to 4 times per week | □ Once or less than once per week | - Never | - I do not own an Amazon Alexa or Google Home |
| --- | --- | --- | --- | --- |

1. **In the last month, have you gone on the Internet or online to order or refill prescriptions?**

| - Yes | - No | - I don’t know | - Prefer not to answer |
| --- | --- | --- | --- |

1. **In the last month, have you gone on the Internet or online to contact any of your medical providers?** (for example, making or changing medical appointments, getting test results, requesting referrals or prescriptions, or to get advice)

| - Yes | - No | - I don’t know | - Prefer not to answer |
| --- | --- | --- | --- |

1. **In the last month, have you gone on the Internet or online to handle Medicare or other health insurance matters?** (for example, going to Medicare's website or another insurer's website to find out what is covered, compare plans or providers, find out about bills, or file a claim)

| - Yes | - No | - I don’t know | - Prefer not to answer |
| --- | --- | --- | --- |

1. **In the last month, have you gone on the Internet or online to get information about your health conditions?**

| - Yes | - No | - I don’t know | - Prefer not to answer |
| --- | --- | --- | --- |

**The next questions ask about how you feel about technology, such as a computer, laptop, tablet or smartphone (such as an iPhone or Android phone).**

1. **I feel comfortable with technology.**

| - Strongly Agree | - Somewhat Agree | - Somewhat Disagree | - Strongly Disagree |
| --- | --- | --- | --- |

1. **Technology makes me nervous.**

| - Strongly Agree | - Somewhat Agree | - Somewhat Disagree | - Strongly Disagree |
| --- | --- | --- | --- |

1. **I don’t feel confident about my ability to use technology.**

| - Strongly Agree | - Somewhat Agree | - Somewhat Disagree | - Strongly Disagree |
| --- | --- | --- | --- |

1. **Technology is confusing.**

| - Strongly Agree | - Somewhat Agree | - Somewhat Disagree | - Strongly Disagree |
| --- | --- | --- | --- |

1. **I feel apprehensive about using technology.**

| - Strongly Agree | - Somewhat Agree | - Somewhat Disagree | - Strongly Disagree |
| --- | --- | --- | --- |

1. **I hesitate to use the technology for fear of making mistakes I cannot correct.**

| - Strongly Agree | - Somewhat Agree | - Somewhat Disagree | - Strongly Disagree |
| --- | --- | --- | --- |

1. **Technology helps me be connected with family and friends.**

| - Strongly Agree | - Somewhat Agree | - Somewhat Disagree | - Strongly Disagree |
| --- | --- | --- | --- |

1. **Technology helps me learn new information and skills.**

| - Strongly Agree | - Somewhat Agree | - Somewhat Disagree | - Strongly Disagree |
| --- | --- | --- | --- |

**When you have problems with your technology, who do you ask for help? (check all that apply)**

| Family member  Friend  Neighbor | Staff at the community where I live  Other: ______________________ |
| --- | --- |

1. **How satisfied are you with the technology help you currently receive from others?**

| - Very Satisfied | - Somewhat Satisfied | - Somewhat Dissatisfied | - Very Dissatisfied |
| --- | --- | --- | --- |

1. **Please use the space below to tell us anything else about yourself or this project.**

________________________________________________________________

________________________________________________________________
